# Supplementary material for: A Global Screen for Assembly State Changes of the Mitotic Proteome by SEC-SWATH-MS
Source: Cell Syst. 2020 Feb 26;10(2):133–155.e6. doi: 10.1016/j.cels.2020.01.001 (PMC7042714; doi:10.1016/j.cels.2020.01.001)

Q9P2E5 | CHPF2_HUMAN | CHPF2 CHSY3 CSGLCAT KIAA1402 UNQ299/PRO339
Monomer MW [kDa]: 85.948 Monomer expected elution fraction: 41

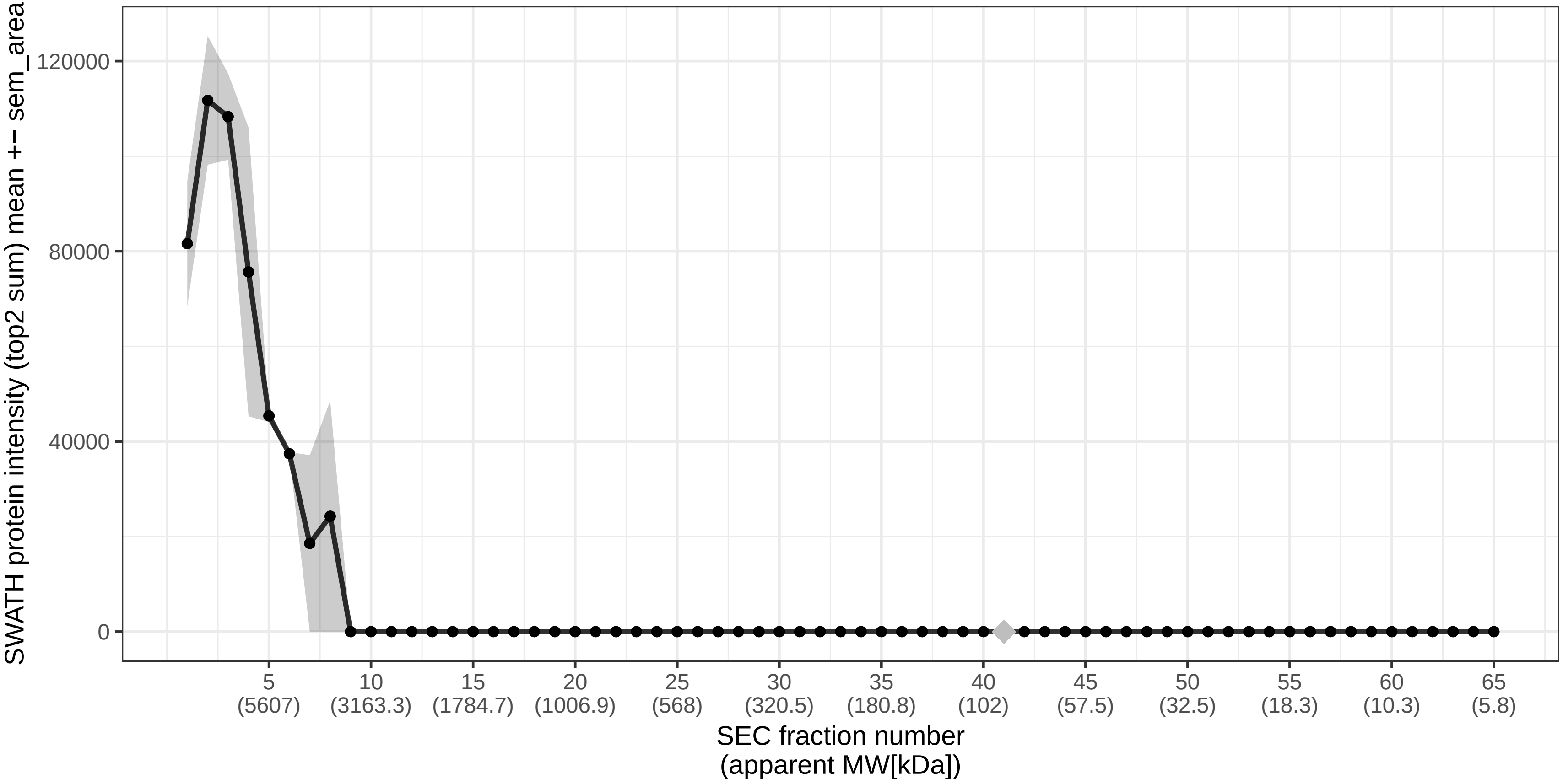

Supplement: Data S1. SEC-SWATH-MS Protein Chromatograms, Related to Figure 1 [file mmc6.zip › SECchrom_Q9P2E5_CHPF2_HUMAN_CHPF2_CHSY3_CSGLCAT_KIAA1402_UN.pdf]
